# Joint Screening for Ultra-High Dimensional Multi-Omics Data

**DOI:** 10.3390/bioengineering11121193

**Published:** 2024-11-25

**Authors:** Ulrich Kemmo Tsafack, Chien-Wei Lin, Kwang Woo Ahn

**Affiliations:** Division of Biostatistics, Medical College of Wisconsin (MCW), Milwaukee, WI 53226, USA; ukemmo@mcw.edu (U.K.T.); chlin@mcw.edu (C.-W.L.)

**Keywords:** variable selection, screening, multi-omics, ultra-high dimensional data

## Abstract

Investigators often face ultra-high dimensional multi-omics data, where identifying significant genes and omics within a gene is of interest. In such data, each gene forms a group consisting of its multiple omics. Moreover, some genes may also be highly correlated. This leads to a tri-level hierarchical structured data: the cluster level, which is the group of correlated genes, the subgroup level, which is the group of omics of the same gene, and the individual level, which consists of omics. Screening is widely used to remove unimportant variables so that the number of remaining variables becomes smaller than the sample size. Penalized regression with the remaining variables after performing screening is then used to identify important variables. To screen unimportant genes, we propose to cluster genes and conduct screening. We show that the proposed screening method possesses the sure screening property. Extensive simulations show that the proposed screening method outperforms competing methods. We apply the proposed variable selection method to the TCGA breast cancer dataset to identify genes and omics that are related to breast cancer.

## 1. Introduction

Investigators often face ultra-high dimensional multi-omics data, where identifying significant genes and omics within a gene is of interest. For instance, identifying the genes and the omics that are significantly associated to the progression of breast cancer might be of interest. This question can be answered using multi-omics data for breast cancer which can be downloaded from the TCGA website. Multi-omics data have tri-level structure: (i) highly correlated genes form a cluster; (ii) each gene has multi-omics and thus forms a subgroup; and (iii) omics within each gene. There is rich literature on group variable selection. Group bridge [1], elastic net [2], and group SCAD [3] select important variables at both group and within-group level. However, such penalized regression methods could be computationally unstable or inefficient for variable selection under the ultra-high dimensional setting. Thus, Qiu and Ahn [4] and Ahn et al. [5] proposed a two-step procedure for ultra-high dimensional data with group variables as follows: (i) screen group variables with low signal in the first step; and (ii) use existing penalized regression to identify important group variables and within-group variables.

Screening methods are typically based on either marginal models [6,7] or joint models [8,9]. Marginal-model-based screening involves fitting a univariable model for each variable and ranking the variables based on their importance to the outcome, using metrics such as *p*-values. Thus, one can easily use the standard software to implement it in practice. However, as Yang et al. [9] pointed out, marginal screening methods discard the variables which are marginally insignificant, but jointly significant for the outcome. In contrast, joint screening can retain these important variables as it assesses their importance to the outcome based on their joint effects. In addition to these statistical learning methods for variable selection, machine learning-based feature selection was applied to analyze animal motion patterns [10] and diffusion model selection procedures [11]. Several screening methods have been developed for data with grouped variables [4,12,13,14]. However, none of them considered a tri-level hierarchically structured data. Because all of these methods considered bi-level variable selection, they may result in inaccurate variable selection for high dimensional tri-level multi-omics data.

Biological pathways are constructed based on observed interactions among molecules in a cell. Investigators often use such biological pathways as groups of genes when identifying important biological pathways and genes is of interest. However, if investigators are interested in selecting important genes and omics, rather than biological pathways, treating biological pathways as groups of genes may result in inaccurate variable selection due to the following reasons. First, depending on experimental conditions, only a portion of genes within a pathway could be highly correlated while the other genes within the pathway are weakly correlated. On the other hand, two genes from different pathways could be highly correlated. Further, there are multiple sources of biological pathways such as KEGG database [15] and reactome database [16,17]. Thus, it is unclear which resource leads to a better accuracy and efficiency in selecting important genes and omics. Moreover, many of such pathways have overlapping genes. For instance, gene ID4 belongs to the NGF-stimulated transcription pathway, the kidney development pathway and the signaling by NTRKs pathway [16]. Overlapping pathways may cause difficulties in screening unimportant genes. As Ahn et al. [5] suggested, disjoint pathways make screening unimportant variables easier. Last, there are still many unknown biological pathways. Thus, using existing pathways may not fully represent the underlying true pathways. Therefore, it is challenging to use existing pathways as groups of genes when identifying important genes and omics is of interest.

To address these limitations of existing literature, we propose a joint screening method for ultra-high dimensional multi-omic data. After screening unimportant genes, one can use existing variable selection method with the remaining genes to select important genes and omics. Our aim is identifying important genes and omics, not selecting important existing biological pathways. Thus, instead of using existing gene pathways, we propose to cluster the genes based on the data to form groups of correlated genes first. These disjoint clusters of genes better reflect gene correlations in the data than existing pathways, and thus may lead to more accurate gene and omics selection. By treating clusters of genes as group variables, we propose to screen unimportant groups of genes and unimportant genes. As a result, the proposed variable selection method identifies important genes and omics within high-dimensional multi-omics data featuring a hierarchical group structure, which has not been explored in the current literature to the best of our knowledge. In Section 2, we outline three stages for the proposed variable selection method: clustering, joint screening, and applying penalized regression. We perform simulation studies considering high-dimensional multi-omics data with the binary outcome to compare the proposed method with some competing methods in Section 3, where the proposed method retained important genes better than some competing methods. While we demonstrate the performance of the proposed method using binary outcome data, the joint screening approach is also applicable to other key outcome types, including continuous and count outcomes. We apply the proposed method and competing methods to breast cancer data in Section 4 and identify new genes associated with breast cancer progression. We conclude the article with a discussion in Section 5.

## 2. Methods

**Figure 1:**
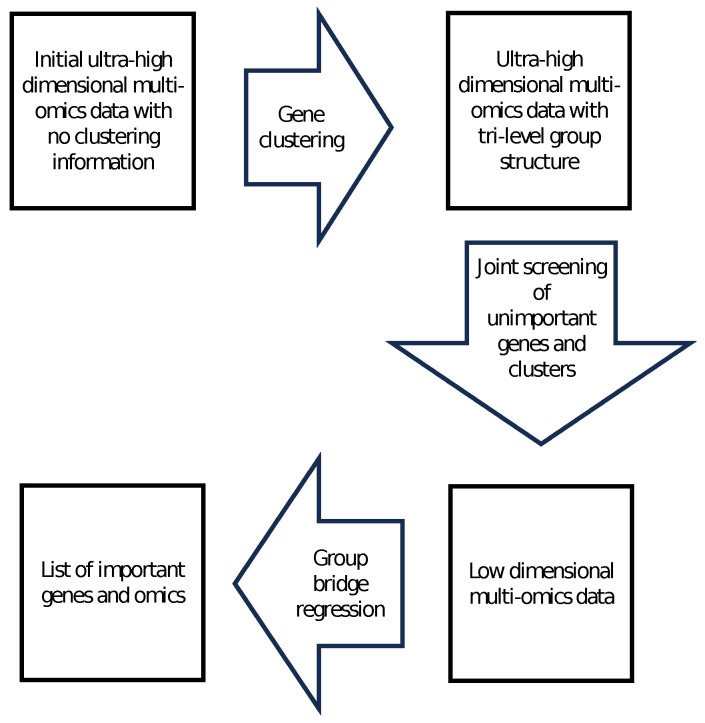
Summary diagram of the proposed variable selection method.

In this section, we describe the clustering algorithm to partition the genes into disjoint groups, such that genes in the same cluster are highly correlated. Then, we propose a joint screening method for ultra-high dimensional multi-omics data. Once we screen unimportant genes, we propose to use group bridge [1] to identify important genes and omics. These steps of the proposed method are illustrated in the diagram of Figure 1.

### 2.1. Clustering Genes with Multi-Omics Data

In this section, we provide a brief description of how to cluster genes with muti-omics data. Weighted correlation network analysis (WGCNA) [18] can cluster genes based on the correlation among genes. The presence of multiple omics within each gene makes it difficult to directly calculate correlations between genes, making WGCNA inapplicable for multi-omics data. Thus, in our previous work, we developed an algorithm called multi-omics meta-analytic gene clustering (MMAGC) [19] that performs gene clustering with multi-omics data. Considering that canonical correlation measures the association between two multivariate sets of vectors, MMAGC uses the canonical correlation to compute the association between two genes for multi-omics data. With the canonical correlation matrix, MMAGC employs a modified WGCNA algorithm to construct gene clusters. In simulation studies, MMAGC outperformed the *K*-means and the traditional WGCNA in gene clustering, where both the *K*-means and the traditional WGCNA use only one type of omics data [19].

### 2.2. Tri-Level Variable Selection

In this section, we propose a variable selection method for ultra-high dimensional multi-omics data. The proposed method selects variables at tri-level: gene-cluster level, gene level, and within-gene level. It consists of two steps: (i) screening unimportant clusters and genes in the first step and (ii) using penalized regression with remaining clusters and genes in the second step. For screening, we consider not only removing unimportant clusters, but also removing unimportant genes within clusters.

#### 2.2.1. Joint Screening for Ultra-High Dimensional Multi-Omics Data

In this section, we propose a joint screening method for tri-level multi-omics data. Let *y* be the outcome of interest. We consider the generalized linear model (GLM) as follows:

(1) $$f\left(E\left(y_{i}|x_{i}\right)\right)=\beta_{0}+x_{i}^{⊤}\beta ,\;\;i=1,\ldots ,n,$$

where *n* is the sample size, $x_{i}$ is a p×1 covariate vector with p≫n, $\beta ={\left(\beta_{1},\ldots ,\beta_{p}\right)}^{⊤}$, and *f* is the link function. Assume the coefficients are sparse and y|x belongs to the exponential family. Thus, the outcome *y* can be continuous, count, and binary. The main purpose of screening is reducing the number of variables to less than *n* while keeping all non-zero genes in the list of the remaining variables; where non-zero genes mean that their coefficients are not equal to zero. The proposed screening method reduces not only the number of clusters, but also the number of genes within a cluster for hierarchically grouped variables.

To elaborate the proposed joint screening method, assume we obtain *C* disjoint gene clusters using MMAGC as in Section 2.1. Let $A_{1},\ldots ,A_{C}$ be a partition of {1,…,p} representing cluster memberships of covariates. Let $\beta_{A_{c}}={\left(\beta_{j},j\in A_{c}\right)}^{⊤}$ be the regression parameters for the covariates in cluster c,c=1,…C and |A| be the number of elements in a set *A*. Without loss of generality, we assume that the $\beta_{j}$’s are on the order of groups such that $\beta^{⊤}=\left(\beta_{A_{1}}^{⊤},\ldots ,\beta_{A_{C}}^{⊤}\right)$, where $\beta_{A_{c}}={\left(\beta_{\alpha_{c}+1},\ldots ,\beta_{\alpha_{c}+\left|A_{c}\right|}\right)}^{⊤}$, $\alpha_{c}=\sum_{i=0}^{c-1}\left|A_{i}\right|$ for c=1,…,C and we define $|A_{0}|=0$. Let $H_{c}$ be the number of genes in cluster *c*. Then, we consider $\beta_{A_{c}}={\left(\beta_{A_{c1}}^{⊤},\ldots ,\beta_{A_{cH_{c}}}^{⊤}\right)}^{⊤}$ where $A_{c}=A_{c1}\cup A_{c2}\cup \ldots \cup A_{cH_{c}}$, c=1,…,C; to emphasize on the gene structure of the cluster. Thus, the total number of genes is $G=\sum_{c=1}^{C}H_{c}$. Assume each gene has $n_{o}$ omics for simplicity. Let l(β) be the log-likelihood function of the model (1). We propose the following joint screening method:

(2) $$\begin{array}{cc} \tilde{\beta }=arg\max_{\beta }l\left(\beta \right)\;subject\;to & 1.\;the\;number\;of\;clusters\;in\;{\left(\beta_{A_{1}}^{⊤},\ldots ,\beta_{A_{C}}^{⊤}\right)}^{⊤}\leq q_{1},\\ \; & 2.\;the\;number\;of\;genes\;in\;\beta_{A_{c}}\leq q_{2c},c=1,\ldots ,C, \end{array}$$

where $q_{1},q_{21},\ldots q_{2C}$ are pre-specified constants. The first constraint ensures to select $q_{1}$ number of clusters. The remaining *C* constraints reduce the number of genes within each cluster. And, $q_{1}$ and the $q_{2c}$’s need to be large enough to include all non-zero variables.

Since p>n, one cannot find a direct solution to Equation (2). To handle this, we approximate l(β) at ω given β with the function g(ω|β) following Ahn et al. [5] as follows:

$$g\left(\omega |\beta \right)=l\left(\beta \right)+{\left(\omega -\beta \right)}^{⊤}l^{\prime }\left(\beta \right)-\frac{u}{2}{\left(\omega -\beta \right)}^{⊤}W\left(\beta \right)\left(\omega -\beta \right),$$

where *u* is a pre-specified large constant and W(β) is a block diagonal matrix from $-l^{\prime \prime }\left(\beta \right)$ as follows:

$$W\left(\beta \right)=\left(\begin{array}{cccc} W_{1}\left(\beta_{A_{1}}\right) & 0 & \ldots & 0\\ 0 & W_{2}\left(\beta_{A_{2}}\right) & \ldots & 0\\ \vdots & \vdots & \ddots & \vdots \\ 0 & 0 & \ldots & W_{C}\left(\beta_{A_{C}}\right) \end{array}\right),$$

$$W_{c}\left(\beta_{A_{c}}\right)=q_{2c}\cdot n_{o}\cdot \left(\begin{array}{cccc} W_{c1}\left(\beta_{A_{c1}}\right) & 0 & \ldots & 0\\ 0 & W_{c2}\left(\beta_{A_{c2}}\right) & \ldots & 0\\ \vdots & \vdots & \ddots & \vdots \\ 0 & 0 & \ldots & W_{cA_{c}}\left(\beta_{A_{cH_{c}}}\right) \end{array}\right),c=1,\ldots ,C,$$

$$W_{ch}\left(\beta_{A_{ch}}\right)=-\left(\begin{array}{ccc} l^{\prime \prime }{\left(\beta \right)}_{\kappa_{c}+1,\kappa_{c}+1} & \ldots & l^{\prime \prime }{\left(\beta \right)}_{\kappa_{c}+1,\kappa_{c}+n_{0}}\\ \vdots & \ddots & \vdots \\ l^{\prime \prime }{\left(\beta \right)}_{\kappa_{c}+n_{0},\kappa_{c}+1} & \ldots & l^{\prime \prime }{\left(\beta \right)}_{\kappa_{c}+n_{0},\kappa_{c}+n_{0}} \end{array}\right),h=1,\ldots ,H_{c},$$

where $l^{\prime \prime }{\left(\beta \right)}_{a,b}$ is the entry (a,b) of the matrix $l^{\prime \prime }\left(\beta \right),\kappa_{c}=\alpha_{c}+n_{0}\left(h-1\right)$, c=1,…,C. Thus, W(β) is a block diagonal matrix, where each block is an $n_{0}\times n_{0}$ matrix and represents the block for the corresponding gene. Note that $W_{ch}\left(\beta_{A_{ch}}\right)$ is the sub-square matrix of $-l^{\prime \prime }\left(\beta \right)$ corresponding to $\beta_{A_{c}h}$, the *h*th gene of the *c*th cluster. Since we use the block diagonal matrix W(β) for screening, we call the proposed screening method as joint screening with the block diagonal matrix (JSBD).

Because all off-block-diagonal elements of W(β) are zero, the solution ω that maximizes g(ω|β) has a closed-form even with p>n. It can be written as $\hat{\omega }=\left({\hat{\omega }}_{A_{1}},\ldots ,{\hat{\omega }}_{A_{C}}\right)=\left({\hat{\omega }}_{A_{11}},\ldots ,{\hat{\omega }}_{A_{1H_{1}}},\ldots ,{\hat{\omega }}_{A_{C1}},\ldots ,{\hat{\omega }}_{A_{CH_{C}}}\right)$ depending on whether we are emphasizing the cluster structure or the gene structure. Our algorithm is based on a bottom-to-top approach. It rejects unimportant genes within a cluster first and then rejects unimportant clusters with selected genes. The clusters are ranked based on their weighted squared sum $R_{c}={\hat{\omega }}_{A_{c}}^{⊤}W_{c}\left(\beta_{A_{c}}\right){\hat{\omega }}_{A_{c}},c=1,\ldots ,C$. The higher the $R_{c}$ value, the more important is the cluster. Similarly, the genes of the same cluster *c* are ranked based on their weighted squared sum $r_{ch}={\hat{\omega }}_{A_{ch}}^{⊤}W_{ch}\left(\beta_{A_{ch}}\right){\hat{\omega }}_{A_{ch}}$, $h=1,\ldots H_{c}$. The following algorithm is used to obtain the solution to Equation (2):

1. Set the initial vector $\beta^{\left(0\right)}=0$.
2. Suppose $\beta^{\left(k\right)}$ is obtained in the *k*th iteration. In the (k+1)th iteration,
  (i) Maximize $g(\omega |\beta^{\left(k\right)})$ and obtain $\hat{\omega }$. Rank genes within each cluster and select the top $q_{2c}$ genes for cluster c,c=1,…,C.
  (ii) Set the following to zero: the value of $\hat{\omega }$ that correspond to the genes non-selected from step 2-(i). Then, obtain the new $\hat{\omega }$. Rank the clusters and select the top $q_{1}$ clusters.
  (iii) Obtain $\beta^{\left(k+1\right)}$ as follows:
    (a) Set the following β’s to zero: the β value of the variables belonging to the clusters that are not in the top $q_{1}$ and the variables belonging to the genes of cluster *c* that are not in the top $q_{2c}$ genes, c=1,…,C.
    (b) Using the remaining non-zero β’s, maximize l(β) to get $\beta^{\left(k+1\right)}$.
3. Repeat step 2 until $\frac{∥\beta^{\left(k+1\right)}-\beta^{\left(k\right)}∥}{∥\beta^{\left(k\right)}∥}<10^{-6}$.

Because $W(\beta^{\left(k\right)})$ is a block diagonal matrix based on genes, we show in Appendix B that Step 2 (i) and (ii) indeed maximize $g(\omega |\beta^{\left(k\right)})$ with the constraints

$$\begin{array}{cc} & 1.\;the\;number\;of\;clusters\;in\;{\left(\omega_{A_{1}}^{⊤},\ldots ,\omega_{A_{C}}^{⊤}\right)}^{⊤}\leq q_{1},\\ & 2.\;the\;number\;of\;genes\;in\;\omega_{A_{c}}\leq q_{2c},c=1,\ldots ,C. \end{array}$$

Next, we study the properties of JSBD. For the parameter estimator $\beta^{\left(k\right)}$ at the *k*th iteration in the optimization algorithm, let

$$\tau^{\left(k\right)}=\underset{\beta }{\sup}\left[\lambda_{max}\left\{W^{-1/2}\left(\beta^{\left(k\right)}\right)\left\{-l^{\prime \prime }\left(\beta \right)\right\}W^{-1/2}\left(\beta^{\left(k\right)}\right)\right\}\right]$$

where $\lambda_{max}\left(M\right)$ denote the largest eigenvalue of the matrix M. Then, we have the following theorem:

**Theorem** **1.** *Assume condition C3 in Appendix A. If $u\geq \tau^{\left(k\right)}$ then $l\left(\beta^{\left(k+1\right)}\right)\geq l\left(\beta^{\left(k\right)}\right)$ in the optimization algorithm.*

The proof of Theorem 1 is provided in Appendix B. It shows the ascent property of the proposed algorithm. It is required to take a large value of *u* to make sure it is greater than $\tau^{\left(k\right)}$ for all *k*. The proposed algorithm does not guarantee that the final estimator $\hat{\beta }$ will converge to the global maximizer. However, the simulation studies in Section 3 show that the algorithm performs well with $u=10^{5}$.

Let $k_{1}$ be true number of non-zero clusters and $k_{2c}$ be the true number of non-zero genes for cluster *c* for c=1,…,C. We assume that $k_{1}\leq q_{1}$ and $k_{2c}\leq q_{2c},c=1,\ldots ,C$. Let $\beta^{*}$ be the true value of β and $s^{*}=\left\{ch:\beta_{A_{ch}}\neq 0\right\}$ be the sets of true non-zero genes. Let $\hat{s}=\left\{ch:{\hat{\beta }}_{A_{ch}}\neq 0\right\}$ be the sets of predicted non-zero genes based on $\hat{\beta }$. For each set of genes s, we denote by ν(s) the set of clusters involved in s; and we denote by $\nu_{A_{c}}\left(s\right)$ the set of genes of group c, (c=1,…,C) involved in s. Let $S_{\left(q_{1},q_{21},\ldots q_{2C}\right)}^{+}=\left\{s:s^{*}\subset s;|\nu \left(s\right)|\leq q_{1};\left|\nu_{A_{c}}\left(s\right)\right|\leq q_{2c},c=1,\ldots ,C\right\}$ and $S_{\left(q_{1},q_{21},\ldots q_{2C}\right)}^{-}=\left\{s:s^{*}⊄s;|\nu \left(s\right)|\leq q_{1};\left|\nu_{A_{c}}\left(s\right)\right|\leq q_{2c},c=1,\ldots ,C\right\}$ be the collection of the over-fitted and the under-fitted models, respectively. JSBD has the sure screening property as follows:

**Theorem** **2.** *Under Conditions (C1) to (C5) in Appendix A, we have $P\left\{\hat{s}\in S_{\left(q_{1},q_{21},\ldots q_{2C}\right)}^{+}\right\}\;\to \;1$ as n→∞.*

The proof of Theorem 2 is provided in Appendix B.

#### 2.2.2. The Group Bridge

After screening clusters and genes with low signal, we use the group bridge regression to select important genes and omics within a gene. Lasso [20] and group lasso [21] select important variables solely at individual level and at group level, respectively. Thus, they do not select variables at group and within-group level. Therefore, we propose to use the group bridge regression [1], which can select important clusters and genes within a cluster.

To elaborate a group bridge approach, let d<n be the number of the retained individual variables after screening. Let $\tilde{C}$ be the number of retained clusters after the screening. Using the retained clusters and genes, the set of indexes of retained clusters can be rearranged in ${\tilde{A}}_{1},\ldots ,{\tilde{A}}_{\tilde{C}}$, disjoint subsets of {1,…,d} representing cluster memberships of genes and omics. The penalized regression with the group bridge penalty is defined as

(3) $$L\left(\beta \right)=-l\left(\beta \right)+\lambda \sum_{c=1}^{\tilde{C}}{\left|{\tilde{A}}_{c}\right|}^{\frac{1}{2}}{\left(\sum_{j\in {\tilde{A}}_{c}}\left|\beta_{j}\right|\right)}^{\frac{1}{2}},$$

where l(β) is the log-likelihood with the retained variables after screening and λ>0 is a tuning parameter. Bayesian information criterion (BIC) or cross validation or generalized cross validation can be used for choosing λ. When fitting the group bridge regression, we treat gene clusters as a group variable and all omics from genes within a cluster as individual variables.

## 3. Simulation and Results

Simulation studies were conducted to compare JSBD with competing methods with the binary outcome. The relationship between a binary outcome and covariates is typically analyzed using logistic regression in practice, due to its popularity and the straightforward interpretation of parameter estimates as odds ratios. Thus, in the simulation studies, three-omics data were generated with a binary outcome of interest using the following logistic regression model:

$$\begin{array}{c} \log\frac{P(Y=1|x)}{1-P(Y=1|x)}=\beta_{0}+x^{⊤}\beta . \end{array}$$

**Figure 2:**
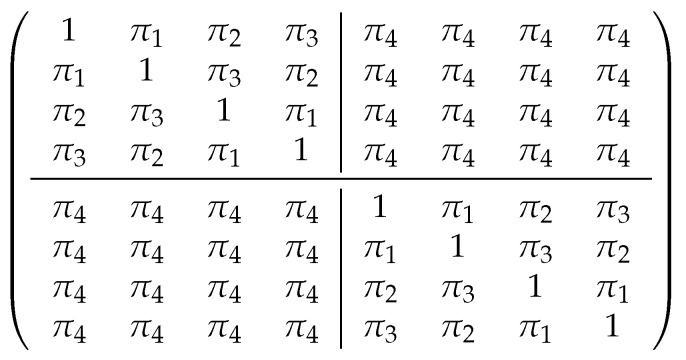
Illustration of the correlation matrix of four genes with two omics each, partitioned into two clusters. The first two genes make the first cluster and the last two genes make the second cluster. The first two columns represent omics 1 and omics 2 of the first gene, the following two columns represent omics 1 and omics 2 of the second gene, followed by omics 1 and omics 2 of the third gene, the last two columns represent omics 1 and omics 2 of the fourth gene.

As illustrated in Figure 2 that shows an example of a correlation matrix of four genes having two omics each, we considered the following four types of correlations:

- correlation between the omics within the same gene: $\pi_{1}$,
- correlation between the same type of omics of different genes in the same cluster: $\pi_{2}$,
- correlation between different types of omics of different genes in the same cluster: $\pi_{3}$,
- correlation between omics from different clusters: $\pi_{4}$.

The covariates were generated using the multivariate normal distribution with mean 0 and the correlation matrix as the covariance matrix. As in our previous work, two scenarios were examined: when $\pi_{1}\geq \pi_{2}$ (scenario 1) and when $\pi_{1}\leq \pi_{2}$ (scenario 2). Four non-zero clusters were considered: 1 cluster of size 5 with 1 non-zero gene with β’s values ${\left(1.5,1.5,1.5\right)}^{⊤}$, 1 cluster of size 10 with 1 non-zero gene with β’s values ${\left(0,0,3\right)}^{⊤}$, 1 cluster of size 20 with 1 non-zero gene with β’s values ${\left(-1.2,-1.4,-1.3\right)}^{⊤}$, and 1 cluster of size 40 with 2 non-zero genes with β’s values ${\left(-2,0,0\right)}^{⊤}$ and ${\left(0,-2.8,0\right)}^{⊤}$. This makes a total of 5 non-zero genes. We considered three sample sizes n=200,400,800 and 200 iterations were conducted. As we wanted the data to remain high-dimensional, we increased *p*, *G*, and *C* with *n*. Their value for each sample size is presented in Table 1. It also shows the number of clusters of size 5, C(5), that of size 10, C(10), that of size 20, C(20) and that of size 40, C(40) considered for each sample size. We set $q_{1}=8$, and the $q_{2c},c=1,\ldots C$ to 10% of the cluster size.

We examined the following competing screening methods in this simulation study:

- The omics-based marginal screening method (OMS) runs the following univariate logistic regression model with each omics and selects the omics with the smallest *p*-values:
  $$\ln\left(\frac{p}{1-p}\right)=\beta_{0}+\beta_{1}Omics,$$
  where Omics is an omics variable.
- The gene-based marginal screening method (GMS) performs a logistic regression model with all the omics of each gene and select the genes with the smallest *p*-values. So, for a three-omics gene, the model is
  $$\ln\left(\frac{p}{1-p}\right)=\beta_{0}+\beta_{1}Omics_{1}+\beta_{2}Omics_{2}+\beta_{3}Omics_{3},$$
  where $Omics_{1},Omics_{2},$ and $Omics_{3}$ are the omics variables of the same gene.
- The proposed joint screening method with diagonal elements (JSD) conducts the proposed joint screening after setting the off-diagonal elements of W(β) to zero. By considering the diagonal elements of W(β), this method ignores the correlation within a gene. We evaluate the importance of the block diagonal matrix for W(β) by comparing JSBD with JSD.

To make the number of retained variables from OMS and GMS consistent with those from JSBD, the number of selected genes in the GMS was set to the number of genes selected by JSBD. The number of omics selected by the OMS was set to the number of genes selected by JSBD times three since each gene had three omics.

We used MMAGC to cluster genes as described in Section 2.1. The adjusted Rand index (ARI) [22] was used to measure the similarity between the underlying true clusters and the predicted clusters. It has zero expected value in the case of random partition and it is bounded above by 1 in the case of perfect agreement between two partitions [22]. Thus, the closer the ARI between the true and predicted clustering is to one, the better it is. For each screening method, we reported (i) the proportion of iterations that all non-zero genes were retained after screening, and (ii) the average proportion of non-zero genes retrained after screening and its standard deviation.

For scenario 1, we considered a very high within gene correlation $\pi_{1}=0.8$ and a strong within cluster correlation $\pi_{2}=\pi_{3}=0.6$. In practice, nearly all genes might be at least weakly correlated. To reflect this into the simulation studies, we set the correlation between omics from different clusters to $\pi_{4}=0.1$. Thus, all omics had a correlation of at least 0.1. The parameter for scenario 2 were obtained by permuting the value of $\pi_{1}$ and $\pi_{2}$ in scenario 1 and get $\left(\pi_{1},\pi_{2},\pi_{3},\pi_{4}\right)=\left(0.6,0.8,0.6,0.1\right)$.

First of all, the ARI between the true clustering and the predicted clustering based on MMAGC was 1 under all settings, indicating that MMAGC predicted the underlying true clusters correctly. Table 2 summarizes the simulation results with the predicted clusters of genes. It includes the proportion of iterations that all non-zero genes are retained after screening, the average proportion of non-zero genes retained after screening, and its standard deviation. Table 2 shows that JSBD kept non-zero genes better than the other competing methods when the sample size is large enough (n≥400). While both JSBD and JSD retained non-zero genes with higher percentages as *n* increased, JSBD led to better variable selection accuracy than JSD. This empirically demonstrated the merit of considering the block diagonal matrix W(β) over the diagonal matrix W(β). On the other hand, the likeliness of marginal screening methods of retaining all non-zero genes showed only very modest improvement with *n*. Both joint screening methods showed higher percentage of containing non-zero genes than the marginal screening methods. We noticed that the performance of each method in scenario 2 was lower than that of the same method obtained in scenario 1. Therefore, more sample size is required when $\pi_{1}\leq \pi_{2}$ to achieve the same performance as when $\pi_{1}\geq \pi_{2}$.

In practice, gene clustering may be inaccurate. Thus, we investigated the robustness against incorrect gene clustering. To do so, we randomly permuted the cluster label of 16% of genes to have some genes belonging to the wrong cluster, resulting in an ARI of 0.719, 0.711, and 0.708 when the sample size was 200, 400 and 800 respectively. With these partially incorrect gene clusters, we applied all the methods for variable screening. Table 3 provides the simulation results. As in the previous simulation study, JSBD retained non-zero genes more effectively than the competing methods when n≥400. The marginal methods are very less likely to select all non-zero genes compare to the joint screening methods. All of these results show that JSBD was the most robust against incorrect gene clustering results. We also noticed that the performance of each method in scenario 2 was lower than that of the same method obtained in scenario 1. Besides, the performance of JSBD and JSD were lower in case of imperfect clustering than that of the same method in case of perfect clustering. This was observed in both scenarios. Therefore, the proposed screening method needs more sample size in case of imperfect clustering to perform as well as it does when the clusters of correlated genes are well identified.

## 4. Real Data Analysis

In this section, we analyzed human breast cancer data downloaded from the TCGA website, project TCGA-BRCA. The aim of the analysis is to identify the genes that are significantly associated with breast cancer. The R package TCGAbiolinks [23,24] was used to download two types of omics data: the RNA sequencing (RNA-seq) count data and the DNA methylation Beta-value data from the “Illumina Human Methylation 27” platform; as well as patients clinical information in the “BCR XML” format. The disease stage was the outcome of interest. We dichotomized the disease stage based on the severity of breast cancer as follows: “0” for stage 1, 1A, 1B, 2, 2A and 2B, and “1” for stage ≥3. The data cleaning and pre-processing include:

- Removing the genes with missing values;
- Matching the ensemble gene ID version to the HUGO gene symbol in the RNA dataset;
- Taking the average of duplicated samples if the average correlation of duplicates is higher than the average correlation of non-duplicate samples; otherwise, randomly selecting one of the duplicates;
- Taking the gene symbol with the highest coefficient of variation among duplicated gene symbols;
- Matching the probe ID to the HUGO gene symbol in the methylation dataset using the Illumina annotation file “HM27.hg38.manifest.gencode.v36.tsv” (downloaded from https://zwdzwd.github.io/InfiniumAnnotation#human, accessed on 15 September 2024);
- Taking the first listed gene symbol when the probe ID was associated with several genes;
- Taking average of methylation beta values across all CpG sites within the same gene;
- Normalization by applying the variance stabilizing transformation (implemented by vst function in R package DESeq2 [25]) to the RNA-seq count data;
- Keeping the samples that have both omics and getting rid of the genes with zero expression level in all those samples;
- Taking the average of repeated samples per patient;
- Removing patients with missing disease stage.

After pre-processing, we had 308 patients and 947 genes, that is, n=308 and p=1894. The clustering algorithm MMAGC led to 20 distinct clusters whose distribution is presented in Table 4. There were 1 cluster of size 461, 112, 86, 74, 42, 36, 30, and 28; 4 clusters of size 11, 2 clusters of size 9, 2 clusters of size 6, and 4 clusters of size 1.

For JSBD and JSD, we set $q_{1}$ and $q_{2c}$ to 10% of the number of clusters and 10% of the cluster size, respectively. For GMS, we kept the same number of genes selected by JSBD. For OMS, we set the the number of retained omics to twice the number of selected genes by JSBD. For the group bridge regression, we chose the tuning parameter λ that minimizes the BIC.

**Figure 3:**
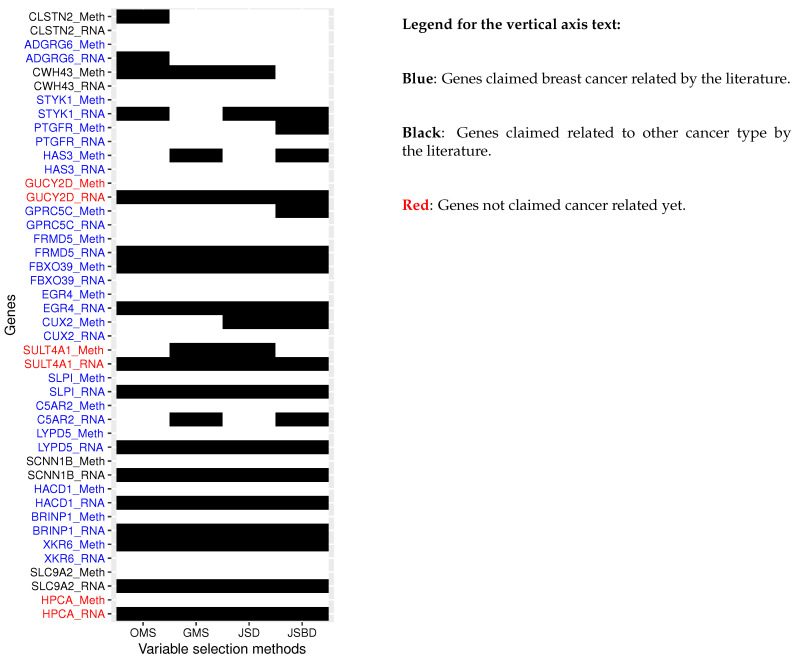
Heatmap summarizing the genes and omics selected by each variable selection method. Each row represents an omics and each column a variable selection method. Black rectangles represent the omics selected by the corresponding variable selection method whereas white rectangles are the omics that are not selected by the corresponding variable selection method.

The omics selected by each variable selection method are summarized in the heatmap shown in Figure 3 where each row represents an omics and each column a variable selection method. Black rectangles represent the omics selected by the corresponding variable selection method whereas white rectangles show the omics that are not selected by the corresponding variable selection method. Omics names are written in the format “Gene name_Omics type”. We extensively investigated whether selected genes are known to be associated with breast cancer, any other type of cancer, or have never been found to be related to breast cancer or other type of cancer. The genes in blue are the ones that have been claimed to be related to breast cancer by the existing literature [26,27,28,29,30,31,32,33,34,35,36,37,38,39,40,41,42,43,44,45]. The genes in black are the ones that have not been claimed to be related to breast cancer, but have been claimed to be related to other type of cancer [46,47,48,49]. The genes in red are the ones that are not known to be related to cancer. Among the genes selected by at least one variable selection method, JBSD missed one gene (ADGRG6) related to breast cancer whereas JSD, GMS and OMS missed 5 genes related to breast cancer. This shows that the variable selection method with JSBD selects more genes related to the progression of breast cancer. HPCA, SLC9A2, SCNN1B, SULT4A1, and GUCY2D selected by the variable selection method with JSBD, that have not been identified by the literature as breast cancer related were also selected by all the other variable selection methods. Thus, these 5 genes may be associated with breast cancer progression. SLC9A2 and SCNN1B which are known related to other type of cancer might have been selected due to the metastasis that happens in the breast cancer late stages. Therefore, the other 3 genes (HPCA, SULT4A1, and GUCY2D) might be related to breast cancer progression either directly or indirectly by being related to other cancer type. Further biological investigations are needed to confirm our discovery. Currently, it is known that HPCA, SULT4A1, and GUCY2D are related to dystonia [50], brain diseases [51,52], and retinal diseases [53,54] respectively. Extensive investigations were done on omics but we could not find any result from the literature. More biological experiments need to be carried out to validate our results on omics. Table 5 summarizes the genes selected by the proposed JSBD variable selection method. As shown in the heatmap, this method identified genes known to be associated with breast cancer, genes associated with other cancer types, and genes not yet linked to cancer. Thus, JSBD identified SLC9A2, SCNN1B, HPCA, SULT4A1, and GUCY2D as new breast-cancer-progression-related genes.

Further, we investigated whether the genes not selected by each variable selection method but selected by others, were retained by the corresponding screening method. Table 6 partitions the genes not selected by each variable selection method into whether they were retained or not retained by the corresponding screening method. The genes in blue are the ones known to be breast cancer related and those in black are the one known to be related to other cancer type. Note that all the genes in the table are cancer related. While JSBD did not retain one breast cancer-related gene, the OMS, GMS and JSD did not retain 2, 5, and 3 breast cancer-related genes, respectively. This suggests that JSBD failed to retain lesser genes related to the outcome variable compared to the competing screening methods.

To evaluate the prediction accuracy of the final model based on each variable selection method, we performed a 5-fold cross-validation 20 times. For each iteration, 4-fold were used for the training and the other fold was used as the testing set to compute the AUC. The mean AUC and its standard deviation as well as the mean number of selected genes and its standard deviation are reported in Table 7. Even though the testing AUC of all the methods are similar, the variable selection with JSBD and the group bridge has the highest AUC. Note that JSBD averagely selects less genes than the JSD and OMS, but still have the highest testing AUC. This indicates that JSBD selected more significant genes compare to JSD and OMS.

## 5. Discussion

Researchers are often focused on identifying important genes and omics in high-dimensional multi-omics data for cancer-related studies. These high-dimensional multi-omics data have a hierarchical group structure, consisting of gene clusters and multi-omics data within each gene. Existing methods do not consider a hierarchical group structure in variable selection. To address this practical need, we have proposed a three-step variable selection method for the tri-level ultra-high dimensional multi-omics data. In the simulation studies, JSBD retained more true non-zero genes than the competing methods, even when gene clustering is inaccurate. When applied to the TCGA human breast cancer data, our proposed method achieved the highest prediction accuracy compared to all other methods we implemented. Based on our extensive search, it also identified several new potential genes associated with breast cancer progression, including HPCA, SLC9A2, SCNN1B, SULT4A1, and GUCY2D. This new knowledge can be further studied by clinicians to further investigate the progression of breast cancer. In the future, this gene list could potentially contribute to the development of a biomarker kit for diagnosing breast cancer progression.

In addition to TCGA where we downloaded our breast cancer data example, investigators can apply our algorithm to data download from other public databases such as gene expression omnibus (GEO), Expression Atlas, Genomic Data Commons (GDC) and cBioPortal.

Investigators often have prior knowledge on a certain important set of variables. In this case, it is of interest evaluating covariates’ conditional contributions to the response given the known set of variables [55]. One can add the group $A_{-1}$ of prior variables to the model and retain $\beta_{A_{-1}}$ when solving Equations (2) and (3). Conducting extensive simulation study for such conditional joint screening is worthwhile in the future. Missing data are common in multi-omics data. Developing a variable selection method for ultra-high dimensional multi-omics data with missing omics would be an important research problem to explore in the future.

It is often of interest evaluating the impact of omics, gene, or gene pathways on the outcome under the high-dimensional multi-omics data. Statistical inference under the high-dimensional setting has recently gotten much attention [56,57,58]. Most existing methods use lasso to handle p>n. However, as Mitra and Zhang [59] pointed out, using group lasso may improve the accuracy of statistical inference on the parameters of group variables. It is worthwhile to develop a statistical inference method based on the penalty function that accounts for hierarchical group structures, such as those found in multi-omics data.

## Figures and Tables

**Table 1:** Covariate information for each sample size.

*n*	*p*	*G*	*C*	C(5)	C(10)	C(20)	C(40)
200	900	300	22	10	5	4	3
400	1680	560	42	20	10	6	6
800	3000	1000	80	40	20	10	10

**Table 2:** Simulation results with perfect clustering structure.

**SCENARIO 1**				
n=200,p=900,G=300,C=22				
	OMS	GMS	JSD	JSBD
Proportion of retaining all non-zero genes ([0–1])	0.295	0.125	0.390	0.335
Proportion of non-zero genes retained ([0–1])	0.790	0.702	0.843	0.821
(SD)	(0.176)	(0.184)	(0.146)	(0.155)
n=400,p=1680,G=560,C=42				
Proportion of retaining all non-zero genes ([0–1])	0.335	0.195	0.730	0.815
Proportion of non-zero genes retained ([0–1])	0.841	0.784	0.941	0.960
(SD)	(0.133)	(0.151)	(0.102)	(0.087)
n=800,p=3000,G=1000,C=80				
Proportion of retaining all non-zero genes ([0–1])	0.365	0.205	0.945	0.955
Proportion of non-zero genes retained ([0–1])	0.865	0.825	0.989	0.991
(SD)	(0.11)	(0.104)	(0.046)	(0.042)
**SCENARIO 2**				
n=200,p=900,G=300,C=22				
	OMS	GMS	JSD	JSBD
Proportion of retaining all non-zero genes ([0–1])	0.040	0.010	0.035	0.025
Proportion of non-zero genes retained ([0–1])	0.571	0.419	0.538	0.548
(SD)	(0.175)	(0.175)	(0.209)	(0.200)
n=400,p=1680,G=560,C=42				
Proportion of retaining all non-zero genes ([0–1])	0.025	0	0.155	0.225
Proportion of non-zero genes retained ([0–1])	0.580	0.471	0.736	0.780
(SD)	(0.176)	(0.142)	(0.170)	(0.160)
n=800,p=3000,G=1000,C=80				
Proportion of retaining all non-zero genes ([0–1])	0.05	0	0.485	0.655
Proportion of non-zero genes retained ([0–1])	0.549	0.475	0.884	0.926
(SD)	(0.177)	(0.128)	(0.123)	(0.109)

**Table 3:** Simulation results with imperfect clustering structure.

**SCENARIO 1**				
n=200,p=900,G=300,C=22,ARI=0.719				
	OMS	GMS	JSD	JSBD
Proportion of retaining all non-zero genes ([0–1])	0.295	0.140	0.280	0.245
Proportion of non-zero genes retained ([0–1])	0.792	0.712	0.796	0.786
(SD)	(0.172)	(0.183)	(0.160)	(0.161)
n=400,p=1680,G=560,C=42,ARI=0.711				
Proportion of retaining all non-zero genes ([0–1])	0.350	0.215	0.500	0.585
Proportion of non-zero genes retained ([0–1])	0.846	0.792	0.874	0.892
(SD)	(0.134)	(0.147)	(0.144)	(0.131)
n=800,p=3000,G=1000,C=80,ARI=0.708				
Proportion of retaining all non-zero genes ([0–1])	0.360	0.220	0.515	0.610
Proportion of non-zero genes retained ([0–1])	0.867	0.827	0.885	0.910
(SD)	(0.105)	(0.107)	(0.135)	(0.123)
**SCENARIO 2**				
n=200,p=900,G=300,C=22,ARI=0.719				
	OMS	GMS	JSD	JSBD
Proportion of retaining all non-zero genes ([0–1])	0.045	0	0.030	0.030
Proportion of non-zero genes retained ([0–1])	0.576	0.413	0.516	0.534
(SD)	(0.177)	(0.170)	(0.206)	(0.206)
n=400,p=1680,G=560,C=42,ARI=0.711				
Proportion of retaining all non-zero genes ([0–1])	0.030	0	0.165	0.150
Proportion of non-zero genes retained ([0–1])	0.578	0.464	0.633	0.683
(SD)	(0.179)	(0.144)	(0.209)	(0.212)
n=800,p=3000,G=1000,C=80,ARI=0.708				
Proportion of retaining all non-zero genes ([0–1])	0.045	0	0.165	0.355
Proportion of non-zero genes retained ([0–1])	0.537	0.470	0.725	0.794
(SD)	(0.175)	(0.133)	(0.194)	(0.196)

**Table 4:** Clustering results for real data from MMAGC. Distribution of cluster sizes.

Cluster index	1	2	3	4	5	6	7	8	9	10
Cluster size	461	112	86	74	42	36	30	28	11	11
Cluster index	11	12	13	14	15	16	17	18	19	20
Cluster size	11	11	9	9	6	6	1	1	1	1

**Table 5:** Results from the proposed variable selection method with JSBD.

	Selested Genes
	XKR6, BRINP1, HACD1, LYPD5, C5AR2,
Genes known breast cancer related	SLPI, CUX2, EGR4, FBXO39, FRMD5,
	GPRC5C, HAS3, PTGFR, STYK1
Genes known related to other cancer types	SLC9A2, SCNN1B
Genes not yet claimed cancer related	HPCA, SULT4A1, GUCY2D

**Table 6:** Genes not retained by each screening method that was selected by some variable selection method.

Screening Methods	Genes Not Retained
OMS	C5AR2, GPRC5C
GMS	CUX2, GPRC5C, PTGFR, STYK1, ADGRG6
JSD	C5AR2, GPRC5C, ADGRG6, CLSTN2
JSBD	ADGRG6, CLSTN2

**Table 7:** Testing mean AUC and mean number of selected genes.

	OMS	GMS	JSD	JSBD
Mean AUC	0.547	0.541	0.554	0.560
(SD)	(0.081)	(0.077)	(0.096)	(0.097)
Mean number of selected genes	10.950	8.620	13.420	9.000
(SD)	(5.430)	(4.836)	(14.716)	(5.540)

## Data Availability

The R package TCGAbiolinks was used to download two types of omics data: the RNA sequencing (RNA-seq) count data and the DNA methylation Beta-value data from the “Illumina Human Methylation 27” platform; as well as patients clinical information in the “BCR XML” format.

## Appendix A. Conditions of Theorems 1 and 2

Let $H=\max_{1\leq c\leq C}H_{c}$ be the maximum number of subgroups in a group. Let $q_{2}=\max_{1\leq c\leq C}q_{2c}$ be the maximum number of retained genes in a cluster. For any set of genes s, we denote by $\beta_{s}={\left(\beta_{j},j\in A_{ch},c\in \nu \left(s\right),h\in \nu_{A_{c}}\left(s\right)\right)}^{⊤}$ the regression parameters for covariates associated with the genes in s. We denote by ${∥\cdot ∥}_{2}$ the euclidean norm. Since y|x belongs to the exponential family, its density function can be written in the form g(y|x;θ)=exp(θy−b(θ)+ρ(y)) where b(·) and ρ(·) are known functions, b(·) being twice continously differentiable, and θ is called the natural parameter. Therefore, we have $E\left(y|x\right)=b^{\prime }\left(\theta \right)$ and $var\left(y|x\right)=b^{\prime \prime }\left(\theta \right)$ where the prime symbol means derivative.

Below are the regularity conditions referred in Theorems 1 and 2.

(C1) There exists $\omega_{1},\omega_{2}>0$ and $\tau_{1},\tau_{2}\geq 0$ such that $\tau_{1}+\tau_{2}<1/2$, $\min_{c\in \nu \left(s\right),h\in \nu_{A_{c}}\left(s\right)}{∥\beta_{A_{ch}}^{*}∥}_{2}\geq \omega_{1}n^{-\tau_{1}}$ and $|s^{*}\left|<|s\right|\leq \omega_{2}n^{\tau_{2}}$ for any collection of subgroups s such that $\left|\nu \left(s\right)\right|\leq 2q_{1};\left|\nu_{A_{c}}\left(s\right)\right|\leq 2q_{2c},c=1,\ldots ,C$.
(C2) $\ln\left(H^{{\,}^{q_{{\,}_{2}}}}C\right)=O\left(n^{m}\right)$ for some $0\leq m<1-2(\tau_{1}+\tau_{2})$.
(C3) There exists constants $c_{1}>0,\delta_{1}>0$, such that for sufficiently large *n*,
  $$-\lambda_{\min}\left[n^{-1}l^{\prime \prime }\left(\beta_{s}\right)\right]\geq c_{1}$$
  for $\beta_{s}\in \left\{\beta_{s}:{∥\beta_{s^{\prime }}-\beta_{s^{\prime }}^{*}∥}_{2}\leq \delta_{1}\right\}$ and $s\in S_{2(q_{1},q_{21},\ldots q_{2C})}^{+}$, where $\lambda_{\min}\left[\cdot \right]$ denotes the smallest eigenvalue of a matrix.
(C4) There exist $c_{2},c_{3}>0$ such that $|x_{ij}|\leq c_{2}$ and when *n* is sufficiently large,
  $\max_{1\leq j\leq p}\max_{1\leq i\leq n}\left\{\frac{x_{ij}^{2}}{\sum_{i=1}^{n}x_{ij}^{2}\sigma_{i}^{2}}\right\}\leq c_{3}n^{-1}$.
(C5) There exists σ>0 such that $b^{\prime \prime }\left(\cdot \right)\leq \sigma$.

### Explanations of the Assumptions

(C1) This condition imposes that $\beta^{*}$ is sparse and that the minimal $L_{2}—$norm of its sub-group components does not degenerate too fast, so that the signal is detectable in the asymptotic sequence.
(C2) This condition allows that $H^{{\,}^{q_{{\,}_{2}}}}C$ diverges with *n* at up to an exponential rate. This allows *p* to diverge with *n* via H,C, and $q_{2}$.
(C3) Together with (C1), this guarantees the identifiability of $s^{*}$ over s for $\left|\nu \left(s\right)\right|\leq q_{1};\left|\nu_{A_{c}}\left(s\right)\right|\leq q_{2c},c=1,\ldots ,C$.
(C4) This condition puts restriction on the observed value of x. As Xu and Chen [8] stated, this holds naturally for the random designs on x or for fixed designs with appropriate re-scaling operation.
(C5) The second derivative of *b* is bounded.

## Appendix B. Proof of Theorems 1 and 2

**Proof** **of** **Theorem** **1.** For any discrete set D(α) of real numbers indexed on α, we define by $arg\max_{\alpha }^{b}D\left(\alpha \right)$, where *b* is an integer, the set of *b* elements $\left\{\alpha_{1},\ldots ,\alpha_{b}\right\}$ such that $\alpha_{1}=arg\max_{\alpha }D\left(\alpha \right),\alpha_{2}=arg\max_{\alpha \neq \alpha_{1}}D\left(\alpha \right),\ldots \alpha_{b}=arg\max_{\alpha \neq \alpha_{1},\ldots \alpha_{b-1}}D\left(\alpha \right)$. To prove Theorem 1, we have the following lemma: **Lemma** **A1.**
*Let $K=\left\{r_{ch},1\leq c\leq C,\;and\;1\leq h\leq H_{c}\right\}$ be a finite sequence of non-negative distinct real numbers, where C and $H_{c},\left(1\leq c\leq C\right)$ are integers. Consider the following optimization problem:*

$$\begin{array}{cc} Find: & \left\{ \begin{array}{c} A\subseteq \left\{1,\ldots ,C\right\}\;with\;|A|\leq q_{1}\;and\\ B_{c}\subseteq \left\{1,\ldots ,H_{c}\right\}\;with\;\left|B_{c}\right|\leq q_{2c}\;for\;each\;c\in A,\\ such\;that\;F=\sum_{c\in A}\sum_{h\in B_{c}}r_{ch}\;is\;maximized \end{array}\right. \end{array}$$

*where $q_{1}$ and $q_{2c},c\in \left\{1,\ldots ,C\right\}$ are integers.*

*For each c∈{1,…,C}, let $B_{c}^{0}=arg\max_{h}^{q_{2c}}\left\{r_{ch},1\leq h\leq H_{c}\right\}$;*

*let $A^{0}=arg\max_{c}^{q_{1}}\left\{\sum_{h\in B_{c}^{0}}r_{ch},1\leq c\leq C\right\}$, then $A^{0}$ and $B_{c}^{0},c\in A^{0}$ is the solution to the optimization problem.*
**Proof** **of** **Lemma** **A1.** Let A⊆{1,…,C} with $\left|A\right|\leq q_{1}$ and for each c∈A, let $B_{c}\subseteq \left\{1,\ldots ,H_{c}\right\}\;with\;\left|B_{c}\right|\leq q_{2c}$. Since $B_{c}^{0}=arg\max_{h}^{q_{2c}}\left\{r_{ch},1\leq h\leq H_{c}\right\}\forall c\in \left\{1,\ldots ,C\right\}$, then for all c∈A, we have $\sum_{h\in B_{c}}r_{ch}\leq \sum_{h\in B_{c}^{0}}r_{ch}$. Hence,

$\begin{array}{ccc} F=\sum_{c\in A}\sum_{h\in B_{c}}r_{ch} & \leq & \sum_{c\in A}\sum_{h\in B_{c}^{0}}r_{ch}\\ & \leq & \sum_{c\in A^{0}}\sum_{h\in B_{c}^{0}}r_{ch}\;since\;A^{0}=arg\max_{c}^{q_{1}}\left\{\sum_{h\in B_{c}^{0}}r_{ch},1\leq c\leq C\right\}; \end{array}$

which proves Lemma A1. □ Next, we apply Lemma A1 to our optimization problem. Let ${∥\beta ∥}_{0}^{A}$ be the number of non-zero $\beta_{A_{c}}$ and $∥\beta_{A_{c}}{∥}_{0}^{B}$ be the number of non-zero $\beta_{A_{ch}},c=1,\ldots ,C,h\in 1,\ldots H_{c}$. Consider finding the solution for the following optimization problem:

(A1) $$\begin{array}{cc} \tilde{\beta }=arg\max_{\beta }l\left(\beta \right)\;subject\;to & {∥\beta ∥}_{0}^{A}\leq q_{1},\\ \; & ∥\beta_{A_{c}}{∥}_{0}^{B}\leq q_{2c},c=1,\ldots ,C. \end{array}$$

Let ${\tilde{\beta }}^{\left(k+1\right)}$ be the parameter vector obtained at step 2−(iii)−(a). Then, $l\left(\beta^{\left(k+1\right)}\right)\geq l\left({\tilde{\beta }}^{\left(k+1\right)}\right)$ because $l(\beta^{\left(k+1\right)})$ is the maximum of l(β) constrained on the zero-entries of ${\tilde{\beta }}^{\left(k+1\right)}$ are equal to zero. Now we show that

$$\begin{array}{cc} {\tilde{\beta }}^{\left(k+1\right)}=arg\max_{\omega }g\left(\omega |\beta^{\left(k\right)}\right)\;subject\;to & ∥\beta^{\left(k\right)}{∥}_{0}^{A}\leq q_{1},\\ & ∥\beta_{A_{c}}^{\left(k\right)}{∥}_{0}^{B}\leq q_{2c},c=1,\ldots ,C. \end{array}$$

The maximizer of g(ω|β) is $\hat{\omega }=\beta +\frac{1}{u}W{\left(\beta \right)}^{-1}l^{\prime }\left(\beta \right)$. We have

$$\begin{array}{ccc} g(\omega |\beta ) & = & l\left(\beta \right)+\sum_{c=1}^{C}\sum_{h=1}^{H_{c}}{\left(\omega_{A_{ch}}-\beta_{A_{ch}}\right)}^{⊤}l_{A_{ch}}^{\prime }\left(\beta \right)-\frac{u}{2}\sum_{c=1}^{C}\sum_{h=1}^{H_{c}}{\left(\omega_{A_{ch}}-\beta_{A_{ch}}\right)}^{⊤}W_{ch}\left(\beta_{A_{ch}}\right)\left(\omega_{A_{ch}}-\beta_{A_{ch}}\right)\\ & = & -\frac{u}{2}\sum_{c=1}^{C}\sum_{h=1}^{H_{c}}{\left\{\omega_{A_{ch}}-\left(\beta_{A_{ch}}+\frac{1}{u}W_{ch}{\left(\beta_{A_{ch}}\right)}^{-1}l_{A_{ch}}^{\prime }\left(\beta \right)\right)\right\}}^{⊤}W_{ch}\left(\beta_{A_{ch}}\right)\left\{\omega_{A_{ch}}-\left(\beta_{A_{ch}}+\frac{1}{u}W_{ch}{\left(\beta_{A_{ch}}\right)}^{-1}l_{A_{ch}}^{\prime }\left(\beta \right)\right)\right\}\\ & & -{\left(\beta_{A_{ch}}+\frac{1}{u}W_{ch}{\left(\beta_{A_{ch}}\right)}^{-1}l_{A_{ch}}^{\prime }\left(\beta \right)\right)}^{⊤}W_{ch}\left(\beta_{A_{ch}}\right)\left(\beta_{A_{ch}}+\frac{1}{u}W_{ch}{\left(\beta_{A_{ch}}\right)}^{-1}l_{A_{ch}}^{\prime }\left(\beta \right)\right)+a\left(\beta \right)\\ & = & \frac{u}{2}\sum_{c=1}^{C}\sum_{h=1}^{H_{c}}{\hat{\omega }}^{⊤}W_{ch}\left(\beta_{A_{ch}}\right)\hat{\omega }-{\left(\omega_{A_{ch}}-{\hat{\omega }}_{A_{ch}}\left(\beta \right)\right)}^{⊤}W_{ch}\left(\beta_{A_{ch}}\right)\left(\omega_{A_{ch}}-{\hat{\omega }}_{A_{ch}}\left(\beta \right)\right)+a\left(\beta \right), \end{array}$$

where a(β) is a function of β free of ω. Note that the $W_{ch}$ are positive definite as a diagonal block of a positive definite matrix by condition (C3). Therefore, maximizing g(ω|β) is equivalent to finding

$$\left\{ \begin{array}{c} A\subseteq \left\{1,\ldots ,C\right\}\;with\;|A|\leq q_{1}\;and\\ B_{c}\subseteq \left\{1,\ldots ,H_{c}\right\}\;with\;\left|B_{c}\right|\leq q_{2c}\;for\;each\;c\in A,\\ such\;that\;\sum_{c\in A}\sum_{h\in B_{c}}{\hat{\omega }}^{⊤}W_{ch}\left(\beta_{A_{ch}}\right)\hat{\omega }=\sum_{c\in A}\sum_{h\in B_{c}}r_{ch}\;is\;maximized. \end{array}\right.$$

By Lemma A1, we have

$$\begin{array}{cc} {\tilde{\beta }}^{\left(k+1\right)}=arg\max_{\omega }g\left(\omega |\beta^{\left(k\right)}\right)\;subject\;to: & ∥\beta^{\left(k\right)}{∥}_{0}^{A}\leq q_{1},\\ & ∥\beta_{A_{c}}^{\left(k\right)}{∥}_{0}^{B}\leq q_{2c},c=1,\ldots ,C. \end{array}$$

This justified why we used the bottom-to-top approach in our algorithm instead of the top-to-bottom one. By Taylor expansion of $l({\tilde{\beta }}^{\left(k+1\right)})$ near $\beta^{\left(k\right)}$,

$$l\left({\tilde{\beta }}^{\left(k+1\right)}\right)=l\left(\beta^{\left(k\right)}\right)+{\left({\tilde{\beta }}^{\left(k+1\right)}-\beta^{\left(k\right)}\right)}^{⊤}l^{\prime }\left(\beta^{\left(k\right)}\right)+\frac{1}{2}{\left({\tilde{\beta }}^{\left(k+1\right)}-\beta^{\left(k\right)}\right)}^{⊤}l^{\prime \prime }\left(\beta \right)\left({\tilde{\beta }}^{\left(k+1\right)}-\beta^{\left(k\right)}\right),$$

where β lies between ${\tilde{\beta }}^{\left(k+1\right)}$ and $\beta^{\left(k\right)}$. We have

$${\left({\tilde{\beta }}^{\left(k+1\right)}-\beta^{\left(k\right)}\right)}^{⊤}\left\{-l^{\prime \prime }\left(\beta \right)\right\}\left({\tilde{\beta }}^{\left(k+1\right)}-\beta^{\left(k\right)}\right)\leq {\left({\tilde{\beta }}^{\left(k+1\right)}-\beta^{\left(k\right)}\right)}^{⊤}W\left(\beta^{\left(k\right)}\right)\left({\tilde{\beta }}^{\left(k+1\right)}-\beta^{\left(k\right)}\right)\tau^{\left(k\right)}.$$

Since $u\geq \tau^{\left(k\right)}$, we have

$${\left({\tilde{\beta }}^{\left(k+1\right)}-\beta^{\left(k\right)}\right)}^{⊤}l^{\prime \prime }\left(\beta \right)\left({\tilde{\beta }}^{\left(k+1\right)}-\beta^{\left(k\right)}\right)\geq -{\left({\tilde{\beta }}^{\left(k+1\right)}-\beta^{\left(k\right)}\right)}^{⊤}W\left(\beta^{\left(k\right)}\right)\left({\tilde{\beta }}^{\left(k+1\right)}-\beta^{\left(k\right)}\right)u.$$

Thus,

$$l\left({\tilde{\beta }}^{\left(k+1\right)}\right)\geq g\left({\tilde{\beta }}^{\left(k+1\right)}|\beta^{\left(k\right)}\right).$$

Since $∥\beta^{\left(k\right)}{∥}_{0}^{A}\leq q_{1}$ and $∥\beta_{A_{g}}^{\left(k\right)}{∥}_{0}^{B}\leq q_{2c},c=1,\ldots ,C$,

$$g\left({\tilde{\beta }}^{\left(k+1\right)}|\beta^{\left(k\right)}\right)\geq g\left(\beta^{\left(k\right)}|\beta^{\left(k\right)}\right)=l\left(\beta^{\left(k\right)}\right).$$

Hence $l\left(\beta^{\left(k+1\right)}\right)\geq l\left(\beta^{\left(k\right)}\right)$, which proves the ascent property. □

**Proof** **of** **Theorem** **2.** We use the following Lemma A2 of Xu and Chen [8] to prove the sure screening property in Theorem 2: **Lemma** **A2.**
*Let $Y_{i},i=1,\ldots ,n$ be independent random variables from exponential family distributions with natural parameters $\theta_{i}\in \Theta$. Let $\mu_{i}$ and $\sigma_{i}^{2}$ denote the mean and variance of $Y_{i}$, respectively. Let $t_{ni},i=1,\ldots ,n$ be real numbers such that*

$$\sum_{i=1}^{n}t_{ni}^{2}\sigma_{i}^{2}=1,\;\;\max_{1\leq i\leq n}\left\{t_{ni}^{2}\right\}=O\left(n^{-1}\right).$$

*Then, for some positive sequence $d_{n}=o\left(n^{1/2}\right)$ and a sufficiently large n,*

$$P\left(\sum_{i=1}^{n}t_{ni}\left(Y_{i}-\mu_{i}\right)>d_{n}\right)\leq \exp\left(-d_{n}^{2}/3\right).$$

Now we show Theorem 2. Let ${\hat{\beta }}_{s}$ be the unrestricted MLE of β based on the model with covariates in s. To show that $P\left\{\hat{s}\in S_{\left(q_{1},q_{21},\ldots q_{2C}\right)}^{+}\right\}\;\to \;1$ as n→∞, it suffices to show that

(A2) $$P\left\{\max_{s\in S_{\left(q_{1},q_{21},\ldots q_{2C}\right)}^{-}}l\left({\hat{\beta }}_{s}\right)\geq \min_{s\in S_{\left(q_{1},q_{21},\ldots q_{2C}\right)}^{+}}l\left({\hat{\beta }}_{s}\right)\right\}\to 0\;\;\;as\;n\to \infty .$$

For any $s\in S_{\left(q_{1},q_{21},\ldots q_{2C}\right)}^{-}$, define $s^{\prime }=s\cup s^{*}\in S_{2(q_{1},q_{21},\ldots q_{2C})}^{+}$. Consider $\beta_{s^{\prime }}$ such that $∥\beta_{s^{\prime }}\;-\;\beta_{s^{\prime }}^{*}{∥}_{2}=\omega_{1}n^{-\tau_{1}}$ for some $\omega_{1},\tau_{1}>0$. When *n* is sufficiently large, we get $∥\beta_{s^{\prime }}\;-\;\beta_{s^{\prime }}^{*}{∥}_{2}\;\leq \;\delta_{1}$ which satisfies condition C3. By Taylor’s expansion around $\beta_{s^{\prime }}^{*}$, we have:

(A3) $$\begin{array}{ccc} l\left(\beta_{s^{\prime }}\right)-l\left(\beta_{s^{\prime }}^{*}\right) & = & {\left[\beta_{s^{\prime }}-\beta_{s^{\prime }}^{*}\right]}^{⊤}l^{\prime }\left(\beta_{s^{\prime }}^{*}\right)+\frac{1}{2}{\left[\beta_{s^{\prime }}-\beta_{s^{\prime }}^{*}\right]}^{⊤}l^{\prime \prime }\left({\tilde{\beta }}_{s^{\prime }}\right)\left[\beta_{s^{\prime }}-\beta_{s^{\prime }}^{*}\right]\\ \; & \leq & {\left[\beta_{s^{\prime }}-\beta_{s^{\prime }}^{*}\right]}^{⊤}l^{\prime }\left(\beta_{s^{\prime }}^{*}\right)-\frac{1}{2}c_{1}n{∥\beta_{s^{\prime }}-\beta_{s^{\prime }}^{*}∥}_{2}^{2}\;\;using\;C3\\ \; & \leq & \omega_{1}n^{-\tau_{1}}{∥l^{\prime }\left(\beta_{s^{\prime }}^{*}\right)∥}_{2}-\frac{1}{2}c_{1}\omega_{1}^{2}n^{1-2\tau_{1}}\;\;by\;Cauchy-Schwarz\;inequality. \end{array}$$

where ${\tilde{\beta }}_{s^{\prime }}$ lies in the segment $\left[\beta_{s^{\prime }},\beta_{s^{\prime }}^{*}\right]$. Then,

$$\begin{array}{ccc} P\left\{l\left(\beta_{s^{\prime }}\right)-l\left(\beta_{s^{\prime }}^{*}\right)\geq 0\right\} & \leq & P\left\{∥l^{\prime }\left(\beta_{s^{\prime }}^{*}\right){∥}_{2}\geq \frac{1}{2}c_{1}\omega_{1}n^{1-\tau_{1}}\right\}\\ & \leq & \sum_{c\in \nu (s^{\prime })}\sum_{h\in \nu_{A_{g}}\left(s^{\prime }\right)}\sum_{j\in A_{gh}}P\left\{{\left[l_{j}^{\prime }\left(\beta_{s^{\prime }}^{*}\right)\right]}^{2}\geq {\left|s^{\prime }\right|}^{-1}\frac{1}{4}c_{1}^{2}\omega_{1}^{2}n^{2-2\tau_{1}}\right\}, \end{array}$$

where

$$l_{j}^{\prime }\left(\beta_{s^{\prime }}^{*}\right)=\sum_{i=1}^{n}\left[y_{i}-b^{\prime }\left(x_{is^{\prime }}^{⊤}\beta_{s^{\prime }}^{*}\right)\right]x_{ij}=\sum_{i=1}^{n}\left[y_{i}-E\left(y_{i}|x_{i}\right)\right]x_{ij}.$$

Let $t_{ni}=x_{ij}{\left(\sum_{i=1}^{n}x_{ij}^{2}\sigma_{i}^{2}\right)}^{-1/2}$, then $\sum_{i=1}^{n}t_{ni}^{2}\sigma_{i}^{2}=1$. Also, by C4 and C5, we have $\max_{i}\left\{t_{ni}^{2}\right\}\leq c_{3}n^{-1}$ and $\sum_{i=1}^{n}x_{ij}^{2}\sigma_{i}^{2}\leq c_{2}^{2}n\sigma^{2}$. Besides, by C1, $|s^{\prime }|\leq \omega_{2}n^{\tau_{2}}$. Therefore,

(A4) $$\begin{array}{ccc} P\left\{l_{j}^{\prime }\left(\beta_{s^{\prime }}^{*}\right)\geq {\left|s^{\prime }\right|}^{-(1/2)}\frac{1}{2}c_{1}\omega_{1}n^{1-\tau_{1}}\right\} & \leq & P\left\{\sum_{i=1}^{n}t_{ni}\left[y_{i}-E\left(y_{i}|x_{i}\right)\right]>\gamma n^{\left(1/2\right)\left(1-2\tau_{1}-\tau_{2}\right)}\right\}\\ \; & \leq & \exp\left[-\left(\gamma^{2}/3\right)n^{\left(1-2\tau_{1}-\tau_{2}\right)}\right]\;by\;Lemma\;A2, \end{array}$$

where γ can be taken as $\gamma =c_{1}\omega_{1}/\left(c_{2}\sigma \omega_{2}^{1/2}\right)$. Using the same process, we can also show that

(A5) $$P\left\{l_{j}^{\prime }\left(\beta_{s^{\prime }}^{*}\right)\leq -{\left|s^{\prime }\right|}^{-(1/2)}\frac{1}{2}c_{1}\omega_{1}n^{1-\tau_{1}}\right\}\leq \exp\left[-\left(\gamma^{2}/3\right)n^{\left(1-2\tau_{1}-\tau_{2}\right)}\right].$$

(A4) and (A5) imply that

$$\begin{array}{ccc} P\left\{l\left(\beta_{s^{\prime }}\right)\geq l\left(\beta_{s^{\prime }}^{*}\right)\right\} & \leq & |s^{\prime }|\exp\left[-\left(\gamma^{2}/3\right)n^{\left(1-2\tau_{1}-\tau_{2}\right)}\right]\\ & \leq & \omega_{2}n^{\tau_{2}}\exp\left[-\left(\gamma^{2}/3\right)n^{\left(1-2\tau_{1}-\tau_{2}\right)}\right]. \end{array}$$

Therefore, we have

$$\begin{array}{cc} & P\left\{\max_{s\in S_{\left(q_{1},q_{21},\ldots q_{2C}\right)}^{-}}l\left(\beta_{s^{\prime }}\right)\geq l\left(\beta_{s^{\prime }}^{*}\right)\right\}\\ & \leq \sum_{s\in S_{\left(q_{1},q_{21},\ldots q_{2C}\right)}^{-}}P\left\{l\left(\beta_{s^{\prime }}\right)\geq l\left(\beta_{s^{\prime }}^{*}\right)\right\}\\ & \leq C^{q_{1}}\prod_{\alpha =1}^{q_{1}}H_{c_{\alpha }}^{q_{2c_{\alpha }}}\omega_{2}n^{\tau_{2}}\exp\left[-\left(\gamma^{2}/3\right)n^{\left(1-2\tau_{1}-\tau_{2}\right)}\right]\\ & =\exp\left[q_{1}\ln\left(C\right)+\left(\sum_{\alpha =1}^{q_{1}}q_{2c_{\alpha }}\ln\left(H_{c_{\alpha }}\right)\right)+\ln\left(\omega_{2}\right)+\tau_{2}\ln\left(n\right)-\left(\gamma^{2}/3\right)n^{\left(1-2\tau_{1}-\tau_{2}\right)}\right]\\ & \leq \exp\left[q_{1}\ln\left(C\right)+\left(\sum_{\alpha =1}^{q_{1}}q_{2}\ln\left(H\right)\right)+\ln\left(\omega_{2}\right)+\tau_{2}\ln\left(n\right)-\left(\gamma^{2}/3\right)n^{\left(1-2\tau_{1}-\tau_{2}\right)}\right]\\ & =\exp\left[q_{1}\ln\left(H^{{\,}^{q_{{\,}_{2}}}}C\right)+\ln\left(\omega_{2}\right)+\tau_{2}\ln\left(n\right)-\left(\gamma^{2}/3\right)n^{\left(1-2\tau_{1}-\tau_{2}\right)}\right]. \end{array}$$

From C2, there is $c_{0}>0$ such that $\ln\left(H^{{\,}^{q_{{\,}_{2}}}}C\right)\leq c_{0}n^{m}$. Also note that $q_{1}\leq \left|s|<\right|s^{\prime }|<\omega_{2}n^{\tau_{2}}$. Hence,

(A6) $$P\left\{\max_{s\in S_{\left(q_{1},q_{21},\ldots q_{2C}\right)}^{-}}l\left(\beta_{s^{\prime }}\right)\geq l\left(\beta_{s^{\prime }}^{*}\right)\right\}\leq \omega_{2}\exp\left[\tau_{2}\ln\left(n\right)+c_{0}\omega_{2}n^{m+\tau_{2}}-\left(\gamma^{2}/3\right)n^{\left(1-2\tau_{1}-\tau_{2}\right)}\right]=o\left(1\right).$$

Since $l(\beta_{s^{\prime }})$ is concave in $\beta_{s^{\prime }}$ by condition C3, the results in Equation (A6) holds for any $\beta_{s^{\prime }}$ such that $∥\beta_{s^{\prime }}-\beta_{s^{\prime }}^{*}{∥}_{2}\geq \omega_{1}n^{-\tau_{1}}$. For any $s\in S_{\left(q_{1},q_{21},\ldots q_{2C}\right)}^{-}$, let ${\tilde{\beta }}_{s^{\prime }}$ be ${\hat{\beta }}_{s}$ augmented with zeros corresponding to the elements in $s^{\prime }\setminus s$. By condition C1, we have $∥{\tilde{\beta }}_{s^{\prime }}-\beta_{s^{\prime }}^{*}{∥}_{2}\geq {∥\beta_{s^{*}\setminus s}^{*}∥}_{2}\geq \omega_{1}n^{-\tau_{1}}$. Therefore,

$$P\left\{\max_{s\in S_{\left(q_{1},q_{21},\ldots q_{2C}\right)}^{-}}l\left({\hat{\beta }}_{s}\right)\geq \min_{s\in S_{\left(q_{1},q_{21},\ldots q_{2C}\right)}^{+}}l\left({\hat{\beta }}_{s}\right)\right\}\leq P\left\{\max_{s\in S_{\left(q_{1},q_{21},\ldots q_{2C}\right)}^{-}}l\left({\tilde{\beta }}_{s}\right)\geq l\left(\beta_{s^{\prime }}^{*}\right)\right\}=o\left(1\right),$$

which proves the sure screening property. □
